# Health-risk behaviours among people with severe mental ill health: understanding modifiable risk in the Closing the Gap Health Study

**DOI:** 10.1192/bjp.2022.143

**Published:** 2023-04

**Authors:** Emily Peckham, Ben Lorimer, Panagiotis Spanakis, Paul Heron, Suzanne Crosland, Lauren Walker, Simon Gilbody

**Affiliations:** Department of Health Sciences, University of York, UK; Department of Psychology, University of Crete, Greece; Department of Health Sciences, University of York, UK and Hull York Medical School, UK

**Keywords:** Severe mental ill health, health risk behaviours, bipolar disorder, schizophrenia, psychosis

## Abstract

**Background:**

People with severe mental ill health (SMI) experience some of the largest health inequalities of any sector within society. For these inequalities to be reduced, an understanding of the behavioural determinants of health in this population is needed.

**Aims:**

Utilising data from the Closing the Gap Health Study, we aimed to assess the extent to which people with SMI report health-risk factors and behaviours, their interest in modifying them, and the factors associated with being motivated to modify these behaviours.

**Method:**

Adult (≥18 years old) participants were recruited via primary and secondary care in the English National Health Service. To be eligible, participants needed to have a documented diagnosis of schizophrenia, psychotic disorders or bipolar disorder. Data were collected by survey on demographics, general physical health, diet, physical activity, alcohol, smoking and body mass index.

**Results:**

Between April 2016 and March 2020, *n* = 9914 participants were recruited. Among people with SMI, high rates of obesity (37.5%), infrequent physical activity (62.0%), not meeting current guidelines (≥5) for the consumption of fruit and vegetables (85.0%) and smoking (42.2%) were observed. However, most participants were motivated to reduce health-risk behaviours. Perceiving the importance of health-promoting behaviours, being of poorer general health and being female were significantly associated with being motivated to modify health-risk behaviours.

**Conclusions:**

Despite experiencing poor physical and mental health outcomes compared with the general population, and contrary to popular misconceptions, people with SMI perceive health as important and are motivated to make behavioural changes to improve health.

## Background

People with severe mental ill health (SMI) die on average 15–20 years earlier than those in the general population,^1–3^ with preventable physical diseases being a significant contributor to this health inequality. For instance, compared with the general population, people with SMI are 2 times more likely to develop diabetes,^4^ 85% more likely to die from coronary heart disease^5^ and 10 times more likely to die from respiratory disease.^6^ Modifiable risk factors, including physical inactivity, poor diet, obesity, smoking, alcohol consumption and the side-effects of psychiatric medication, also contribute to this health inequality. Environmental and economic determinants are also important, considering that people with SMI have limited access to green spaces, and experience financial and social inequalities and adversity.^7,8^ Improving our understanding of the prevalence and extent of these risk factors, and people's willingness to modify them (where possible), will help to guide the development of strategies to reduce the impact of such factors and close the ‘health and mortality gap’.

## Aims

The Closing the Gap Health Study was established in response to these concerns regarding the poor physical health and mortality of people with SMI. Using data from the Closing the Gap Health Study, we aimed to assess the extent to which people with SMI engage in certain health-risk behaviours (such as smoking), and the degree to which these people are interested in modifying them (for example cutting down or quitting smoking). We also investigated factors that are associated with being motivated to modify health risk.

## Method

### Design and setting

This is the first paper reporting data gathered from the Closing the Gap Health Study, which was a cross-sectional survey study established in 2016. Pilot recruitment took place in primary and secondary care sites in the Yorkshire and Humber region of England, and in one secondary care site in South East England. Following a successful 6-month pilot of 500 participants, recruitment was expanded in 2018 to 314 primary care sites and 23 secondary care sites across England, with the aim being to recruit 10 000 participants. Ethical approval was obtained from the West Midlands – Edgbaston Research Ethics Committee (ref: 15/WM/0444)

### Participants

A transdiagnostic approach was adopted to capture the range of conditions collectively known as ‘severe mental ill health’ (SMI).^9^ Eligibility criteria were:

- aged 18 years or over;
- had capacity to consent; and
- had received a documented (clinician allocated) diagnosis of schizophrenia, bipolar disorder or associated disorder (corresponding to ICD-10 F20–29 or F30–31 or DSM-IV 295.x, 296.x, or 297.x criteria).

Participants were recruited from primary and secondary care. In primary care, general practitioner (GP) practices posted an invitation pack to patients on their SMI register that contained a cover letter, a participant information sheet, the study questionnaire and a pre-paid return envelope. Meanwhile in secondary care, clinicians provided eligible patients from their case-load with a participant information sheet and referred them to a local researcher. Potential participants who wished to take part could then choose to complete the survey with a local researcher, with their clinician or by themselves.

### Study questionnaire

The study questionnaire was a self-report survey that took approximately 10 min to complete. Participants were asked 23 questions related to: demographic information, general health, diet, physical activity, alcohol use, smoking and body mass index (BMI). The questions mirrored those previously used by the Office of National Statistics,^10^ therefore enabling study responses to be compared with the general population. The last page of the survey contained a consent form which asked participants: (a) for permission for future contact; (b) to let the participant's GP/ mental health professional know they are taking part in the study; (c) for permission to look at their health records; and (d) for their contact details, should participants choose to provide them. The provision of consent to future contact allows researchers to contact participants in the study and invite them to take part in other research studies that they may be interested in.

### Measures

Sociodemographic characteristics gathered from participants included age, gender, employment status, ethnicity and neighbourhood deprivation. Full details of the measures can be found in Supplementary Appendix 1 available at https://doi.org/10.1192/bjp.2022.143. Neighbourhood deprivation was determined by linking the participants post code with the English Index of Multiple Deprivation (IMD).^11^ Participants were also asked to rate their general health in the past 12 months and whether any health problem limited their activity. In addition, they were asked to self-report their height and weight from which their BMI was calculated. To explore health-risk behaviours participants were asked about; frequency of taking part in physical activity, the number of portions of fruit and vegetables they ate per day and their smoking status. Those who reported smoking were asked how many cigarettes per day they smoked. Motivation to change health-risk behaviours was explored by asking about; motivation to increase levels of physical activity, change diet or reduce weight and cut down or quit smoking. Finally, participants were asked how important they perceived maintaining a healthy lifestyle.

### Patient and public involvement

All the study's materials were reviewed by service users, following which they were adapted considering their comments. The study had a lived experience/service user (and carer) advisory group who provided advice on the running of the study and the interpretation of results.

### Analysis

All analyses were performed using R Statistical Software.^12^ Descriptive statistics were used to profile demographic and health information, perceived health importance, health-risk behaviours and motivations to change. In addition, four binary logistic regression models were conducted, with a Bonferroni-adjusted alpha value of 0.0125 (0.05/4) being adopted. The relevant assumptions of logistic regression (i.e. no multicollinearity, and linearity to the logit) were tested for each of the conducted regressions. Before applying the regression models, non-parametric missing value imputation was performed on the sample using the R package *missForest.*^13^ MissForest is an algorithm based on the machine learning approach of Random Forest, which can impute missing values in mixed-type data-sets (i.e. contains both continuous and categorical variables), and has been demonstrated to be effective at handling missing values in variables that have up to 30% missing information.^14^ All variables were treated as being categorical in nature, excluding age, IMD and level of fruit and vegetable consumption, which were treated as continuous variables. Before imputing missing values, participants who had missing information for at least 50% of variables were excluded.

For the first logistic regression model, the dependent variable was the dichotomous variable of perceived importance of healthy living (‘some importance’ versus ‘no importance’). Seven independent variables were input into this model: age, gender, employment status, ethnicity, IMD, BMI category and self-reported general health. This model was developed using the entire sample.

For the remaining three regression models, the dependent variables were the participants’ motivations to change (‘wanting to change’ versus ‘not wanting to change or unsure’) three specific health-risk behaviours. One model focused on motivation to exercise more, another focused on motivation to change diet or lose weight and the final model focused on motivation to cut down or quit smoking. Each of the three models were developed using a different subsample. The model on exercise motivation was developed using only those participants who were not engaging in physical activity daily or every other day. The model on diet/weight motivation was developed using only those participants who were either overweight, obese or who ate less than five fruit or vegetables per day. Finally, the model on smoking motivation was developed using only those participants who smoked.

Seven independent variables were input into all three motivation models, including: age, gender, employment, ethnicity, IMD, self-reported general health and perceived importance of healthy living. In addition, BMI category and frequency of physical activity were also input as independent variables for the exercise motivation model, whereas the BMI category and being a heavy smoker or not were also input as independent variables for the smoking motivation model. As a sensitivity analysis, all four models were redeveloped using only those participants with complete information (i.e. using non-imputed data).

## Results

### Participants

Between April 2016 and March 2020, *n* = 9914 participants were recruited, of which 3084 were recruited from primary care services and 6830 were recruited from secondary care services.

Table 1 describes the sociodemographic and health information of the participants. Demographic and health information statistics (i.e. means, s.d.s and percentages) were calculated using only those participant with full, relevant information. The mean age was 48.2 (s.d. = 14.8; range = 18–101; interquartile range (IQR) = 37–58), with 55.4% of the participants being men and 85.2% being of White ethnicity. Most participants were not in paid employment (82.6%) and participants mostly lived in neighbourhoods that had high levels of deprivation (mean 4.7; s.d. = 2.8; range = 1–10; IQR = 2–7).

**Table 1:** Sociodemographic and health information of the sample (*n* = 9914)

Variable	Value^a^	Missing *n*
*Sociodemographic information*		
Gender, *n* (%)		180^b^
Men	5388 (55.4)	
Women	4289 (44.1)	
Transgender	57 (0.01)	
Age, mean (s.d.)	48.2 (14.8)	203
Ethnicity, *n* (%)		116
White	8350 (85.2)	
Black	434 (4.4)	
Mixed	333 (3.4)	
South Asian	426 (4.3)	
Other Asian	96 (1.0)	
Other	159 (1.6)	
Employment, *n* (%)		221
In paid employment	1687 (17.4)	
Not in paid employment	8006 (82.6)	
Index of Multiple Deprivation, mean (s.d.)	4.7 (2.8)	2493
*Health information*		
Body mass index, mean (s.d.)	28.9 (6.9)	1310
Body mass index Category, *n* (%)		1310
Underweight	183 (2.1)	
Healthy weight	2538 (29.5)	
Overweight	2657 (30.9)	
Obese	3226 (37.5)	
General health rating, *n* (%)		65
Excellent	687 (7.0)	
Good	2813 (28.6)	
Moderate	3710 (37.7)	
Poor	1938 (19.7)	
Very Poor	701 (7.1)	
Activity impaired by health problem, *n* (%)		670^c^
Yes	5990 (64.8)	
No	3254 (35.2)	
Importance of maintaining a healthy lifestyle, *n* (%)		78
A top priority	4475 (45.5)	
Moderately important	3839 (39.0)	
Not worried about it	1522 (15.5)	

a. Percentages calculated using only those patients with full data (i.e. excluding missing).

b. Total includes participants who responded ‘Prefer not to say’.

c. Total includes participants who responded ‘Don't know’.

In terms of health, 35.5% rated their overall general health as being excellent or good over the previous 12-month period, 37.7% rated their health as being moderate, and 26.8% rated their health as poor or very poor. Participants had an average BMI of 28.9 (s.d. = 6.86; range = 14.5–75.1; IQR = 24.0–32.6), with 68.4% being overweight or obese, and 64.8% of the participants reported that a health problem had an impact on their activity. Finally, 45.5% of participants said that maintaining a healthy lifestyle was a top priority for them, whereas 39.0% said that it was moderately important, and 15.5% said it was not something they worried about.

### Health-risk behaviours and motivations to change

**Fig. 1:**
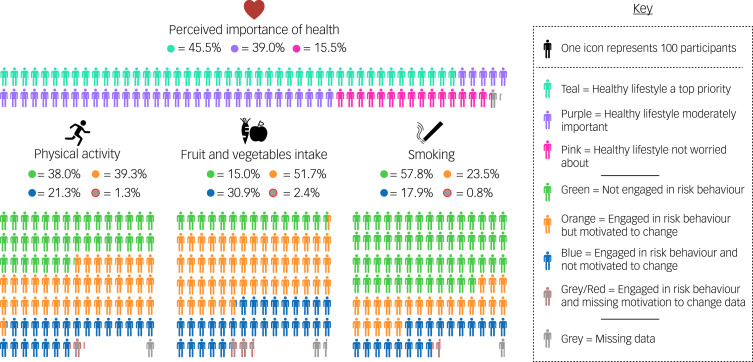
Proportions of participants who perceived health as important, engaged in health-risk behaviours and wanted to reduce engagement in these behaviours. Note: Percentages were calculated using only those participants with recorded response for perceived importance of health/engagement in behaviours (i.e. excluding missing). The number of missing values for the four variables were: perceived importance of health *n* = 78; physical activity *n* = 96; fruit and vegetables intake *n* = 155; and smoking *n* = 72. For ‘fruit and vegetables intake’, the risk behaviour displayed refers to consuming less than five fruit or vegetables per day, and the motivation to change behaviour displayed refers to wanting to ‘change diet or lose weight’.

Figure 1 illustrates the extent of the participants’ engagement in the three health-risk behaviours, and the proportions of participants who reported wanting to modify their engagement in these behaviours. The percentages noted within the figure were calculated using only those participants with a recorded response for the relevant variable (i.e. excluding missing values). Just under two-thirds of participants (62.0%) reported that they did not engage in physical activity daily or every other day, with almost one-quarter (23.5%) reporting that they never engaged in physical activity. Of those participants engaging in physical activity less than daily or every other day, 64.8% reported that they wanted to exercise more.

For diet, 85.0% of participants reported that they did not eat five or more portions of fruit and vegetables per day, and 8.4% reported not eating any portions per day. The participants reported eating a mean of 2.5 (s.d. = 1.5) fruit and vegetables per day. Of those participants who did not meet current guidelines for the consumption of fruit and vegetables, or were overweight or obese, 63.3% reported that they wanted to either change their diet or lose weight.

Finally, 42.2% of participants reported that they currently smoked, with 38.1% of these participants being heavy smokers. In terms of motivation to change, 56.7% of smoking participants reported that they wanted to either cut down or quit smoking.

### Regression models

#### Data pre-processing

Before imputing missing values, 17 participants were excluded as they had more than 50% missing information across variables. All variables had less than 5% missingness, except for IMD and BMI, which had 25.1% and 13.2% missingness, respectively. However, as both proportions were less than 30%, both variables were included in the imputation.

Following imputation, transgender participants were excluded from further analyses, because of there being too few participants of this gender within the imputed data-set (*n* = 57; 0.006%). Data pre-processing therefore resulted in a final imputed sample of *n* = 9840 that was available for regression analyses. Finally, it was found that the variable of age violated the assumption of linearity to the logit for the regression models investigating motivation to change diet or lose weight, and motivation to cut down or quit smoking. Therefore, the quadratic polynomial of age was included in each model, as this enabled the assumption to be satisfied. All other assumptions were satisfied.

#### Perceived importance of health

Results of the regression analysis investigating factors associated with perceived importance of health can be found in Table 2. For the full sample of *n* = 9840, it was found that participants were significantly more likely (all *P* < 0.01) to perceive maintaining a healthy lifestyle as important if they were female, were Black and minority ethnic, had an excellent/good self-reported rating of their overall general health, were in paid employment, or lived in less deprived neighbourhoods. In a sensitivity analysis in which only participants with complete information were included, similar findings were identified, except that ethnicity no longer had a significant association (Supplementary Table 1).

**Table 2:** Factors associated with importance of maintaining a healthy lifestyle (*n* = 9840)

Variable	Odds ratio (95% CI)	*P*
Gender		
Men	1	
Woman	1.41 (1.26–1.59)	<0.001*
Age	1.00 (0.99–1.00)	0.399
Ethnicity		
White	1	
Black and minority ethnic	1.44 (1.22–1.72)	<0.001*^a^
Employment		
In paid employment	1	
Not in paid employment	0.63 (0.53–0.76)	<0.001*
Index of Multiple Deprivation	1.11 (1.08–1.14)	<0.001*
Body mass index		
Healthy weight	1	
Underweight	0.68 (0.48–0.98)	0.035
Overweight	1.05 (0.91–1.21)	0.528
Obese	1.00 (0.87–1.14)	0.945
General health rating		
Excellent/Good	1	
Moderate	0.81 (0.71–0.93)	0.004*
Poor/Very Poor	0.50 (0.44–0.58)	<0.001*

a. Ethnicity not statistically significant in sensitivity analysis (regression model with complete case data only; see Supplementary Table 1).

* Statistically significant when tested against a Bonferroni-adjusted alpha value of 0.0125.

#### Motivations to change behaviours

Results from the three regression models that investigated factors associated with being motivated to take more exercise, change diet or lose weight, or cut down or quit smoking can be found in Table 3.

**Table 3:** Factors associated with motivation to take more exercise (*n* = 6111), change diet or lose weight (*n* = 9344) or cut down or quit smoking (*n* = 4130)

	Take more exercise		Change diet/lose weight		Cut down/quit smoking	
Variable	Odds ratio (95% CI)	*P*	Odds ratio (95% CI)	*P*	Odds ratio (95% CI)	*P*
Gender						
Men	1		1		1	
Women	1.36 (1.21–1.53)	<0.001*	1.99 (1.81–2.18)	<0.001*	1.14 (1.00–1.31)	0.050
Age, years	0.98 (0.98–0.99)	<0.001*	1.05 (1.03–1.07)	<0.001*	1.02 (0.99–1.05)	0.098
Age^2^, years^a^	−	−	1.00 (1.00–1.00)	<0.001*	1.00 (1.00–1.00)	0.038
Ethnicity						
White	1		1		1	
Black and minority ethnic	1.02 (0.87–1.19)	0.853	0.90 (0.80–1.04)	0.111	1.07 (0.90–1.27)	0.474
Employment						
In paid employment	1		1		1	
Not in paid employment	0.54 (0.45–0.65)	<0.001*	0.83 (0.73–0.94)	0.003*^b^	0.89 (0.73–1.08)	0.234
Index of Multiple Deprivation	1.06 (1.04–1.09)	<0.001*^b^	1.02 (1.00–1.04)	0.033	1.00 (0.97–1.03)	0.915
General health rating						
Excellent/good	1		1		1	
Moderate	1.14 (0.99–1.30)	0.070	1.43 (1.29–1.58)	<0.001*	1.26 (1.09–1.47)	0.003*^b^
Poor/very poor	1.37 (1.18–1.59)	<0.001*	1.55 (1.38–1.73)	<0.001*	1.40 (1.19–1.65)	<0.001*
Importance of healthy lifestyle						
No importance	1		1		1	
Some importance	2.74 (2.39–3.15)	<0.001*	1.88 (1.68–2.12)	<0.001*	1.78 (1.52–2.08)	<0.001*
Body mass index						
Healthy weight	1		−	−	1	
Underweight	1.16 (0.79–1.72)	0.450	−	−	0.69 (0.46–1.03)	0.069
Overweight	1.45 (1.25–1.68)	<0.001*	−	−	1.16 (0.99–1.36)	0.071
Obese	1.84 (1.60–2.11)	<0.001*	−	−	1.26 (1.09–1.47)	0.003*^b^
Frequency of physical activity						
Weekly	1		−	−	−	−
Less than weekly	1.04 (0.89–1.21)	0.640	−	−	−	−
Never	0.56 (0.49–0.64)	<0.001*	−	−	−	−
Heaviness of smoking						
≥20 cigarettes per day	−	−	−	−	1	
<20 cigarettes per day	−	−	−	−	1.13 (0.99–1.29)	0.080

a. The quadratic polynomial of age was include in the models investigating motivation to change diet/lose weight and cut down/quit smoking, as the variable of age was found to violate the assumption of linearity to the logit for these models.

b. Variable not statistically significant in sensitivity analysis (regression models with complete case data only; see Supplementary Table 1).

* Statistically significant when tested against a Bonferroni-adjusted alpha value of 0.0125 (0.05/4).

For *n* = 6111 participants who were not engaging in physical activity at least daily or every other day, wanting to take more exercise was significantly associated with perceiving a healthy lifestyle as being important, being obese or overweight (compared with healthy weight), being in paid employment, being female, or having a poor/very poor self-reported rating of overall general health (compared with having a good/very good rating). In addition, participants who never engaged in physical activity were significantly less likely to want to take more exercise. It was also found that participants who were younger or living in less deprived neighbourhoods were more likely to want to take more exercise; however, the effect sizes for these two associations were small, and IMD was also no longer significant when only participants who had complete information were investigated (Supplementary Table 2).

For *n* = 9344 participants who did not eat at least five fruit or vegetables per day or were overweight or obese, it was found that participants were significantly more likely to want to change diet or lose weight if they were female, perceived a healthy lifestyle as being important, or had a moderate or poor/very poor self-reported rating of overall general health. It was also found that employment and the quadratic polynomial of age were significantly associated with the outcome; however, both associations had small effect sizes, and employment was not significant when only those participants who had no missing data were investigated (Supplementary Table 2).

Finally, for *n* = 4130 participants who smoked, wanting to cut down or quit smoking was significantly associated with perceiving maintaining a healthy lifestyle as being important, or having a poor/very poor self-reported rating of overall general health (compared with having a good/very good rating). Being obese and having a moderate rating of general health were also found to be significantly associated with the outcome; however, the effect sizes for both variables were small, and both variables were not significant when only investigating those participants who had complete information (Supplementary Table 3).

## Discussion

Using data from the Closing the Gap Health Study, we explored the extent to which people with SMI describe or engage in health-risk behaviours. By examining data from *n* = 9914 participants, we found that a large proportion engaged in various health-risk behaviours. For example, 62.0% of participants reported that were not physically active at least every other day, compared with 33% in the general population.^15^ In addition, only 15.0% of participants consumed the recommended daily number of fruit and vegetables and 37.5% of participants were found to be obese, compared with 28% in the general population for both statistics.^15^ Furthermore, 42.2% of the participants reported being current smokers, almost triple that of the general population.^16^ Combined, these figures indicate that people with SMI are at greater risk of developing long-term physical health problems because of their increased engagement in modifiable health-risk behaviours.

Encouragingly, however, most of the sample (84.5%) perceived maintaining good health as being somewhat important. Two factors identified as being significantly associated with perceiving health as important were being female and being in paid employment. Unemployment being an important factor for perceiving health as being unimportant is concerning, considering that most of the sample were not in paid employment (82.6%). In addition, perceiving health as being unimportant was associated with having a poorer self-reported rating of general health. Further research is required to examine the direction of effect between these two variables.

An encouraging finding was that, of those participants who engaged in specific health-risk behaviours, the majority were motivated to change their engagement in these behaviours. For example, 56.7% of participants who smoked reported wanting to cut down or stop smoking, 63.3% who did not meet current guidelines for fruit and vegetable consumption and/or were overweight/obese reported that they wanted to change their diet or lose weight. In comparison, recent reports estimated that 52.7% of people in the general population want to quit smoking, and 47% would like to lose weight.^15^ This suggests that although people with SMI engage in health-risk behaviours at a higher level compared with the general population, they are just as likely to want to modify health risk to improve their health.^17^ It was also identified that participants who perceived maintaining a healthy lifestyle as important were significantly more likely to be motivated to modify health risk than those participants who perceived health as unimportant. Indeed, this was consistently observed across all three investigated behaviours, with perceived importance of health being the strongest risk factor for motivations to increase exercise and cut down smoking, and the second strongest for motivation to change diet or lose weight. This suggests that health beliefs are important as they may facilitate people with SMI to want to reduce their engagement in health-risk behaviours. Consequently, interventions designed to both promote and provide education related to the importance of health and health behaviours may help facilitate people with SMI to be motivated to reduce their engagement in health-risk behaviours.

In addition, motivation to reduce such behaviours was consistently associated with poorer self-reported general health, indicating that experiencing poor general health is associated with wanting to modify health risk. However, participants who engaged in health-risk behaviours, but had better general health, were less likely to be motivated to change these behaviours, which may indicate that people with SMI are more likely to become motivated after they start to experience physical health problems. Considering these behaviours are associated with longer-term poor physical health outcomes, it is critical to emphasise the importance of prevention and education regarding long-term health consequences of engaging in health-risk behaviours.

Other factors were identified as being significantly associated with motivation to change behaviours. For example, being female was significantly associated with wanting to take more exercise and change diet, but not quitting smoking. Meanwhile, people who never engaged in physical activity were significantly less likely to want to exercise more. This is highly concerning and indicates a need for interventions to facilitate motivation to increase physical activity in this group, as it is highly unlikely that those who do not express a desire to increase physical activity are going to make a change from being inactive to becoming active.^18^

Current knowledge regarding effective strategies to motivate those not currently motivated to change health-risk behaviours is limited and requires further investigation. However, it has been suggested that targeting self-efficacy, outcome expectancies, effort and value beliefs, and using motivational interviewing techniques may prove beneficial^19^ when facilitating motivation to change. Within the population of people with SMI, such techniques could potentially be targeted to men, individuals who perceive health as unimportant and individuals who have better physical health, as these groups may require the most support to increase their motivation to become healthier.

Finally, although noting the individual barriers and attitudinal enablers to behavioural-risk modification, we note that people with SMI often experience ‘therapeutic nihilism’ for behaviours such as smoking, meaning that effective interventions are not offered. This is particularly true for people with multiple long-term health problems in the presence of SMI.^20^ Practice and policy in relation to mental health and the management or prevention of long-term health problems can potentially be informed by this study's findings. At present, physical care is limited to the (often suboptimal) management of health problems such as diabetes and ischaemic heart disease, and preventive strategies could better focus on the management of behavioural-risk factors.^21^ There is clear evidence regarding ‘what works’ in managing such factors,^22^ and the present study provides evidence of the willingness to engage in behavioural health programmes and indicates those populations where willingness to engage is highest.

### Strengths and limitations

To our knowledge this is the largest population study examining physical health and health risk among people with SMI in England. Participants were recruited using a mixture of recruitment methods from a wide range of areas across England in both rural and urban settings and from both primary and secondary care. This allowed for the capture of a broad range of experiences. Furthermore, the composition of the sample aimed to be representative of the investigated population. For example, the proportion of participants who were White (85.2%) was highly similar to the proportion of people in the 2011 Census who were both White and reported as having schizophrenia or schizoaffective disorders (86%).^23^ Participants were asked for consent to be contacted for future research, allowing for them to be recruited to other studies. This enabled a subsample of participants to be quickly recruited to participate in a longitudinal study exploring the impact of the COVID-19 pandemic on people with SMI.^24,25^

However, the study also had several limitations. For example, there may have been a ‘healthy population’ effect whereby healthier individuals were more likely to participate. To mitigate against this, many participants were recruited from places in which they were more likely to be unwell, such as clozapine clinics and in-patient wards. Moreover, the survey question that asked participants about their motivation to change their diet also asked about motivation to lose weight. As a result of this, it was not possible to distinguish whether participants were motivated to change their diet or lose weight (or both), and to determine if associated factors were different for the two outcomes. Future research should aim to separate these two motivations to enable more specific analyses. Furthermore, another limitation is that participants were only recruited in England, and therefore results may not generalise to other countries in the UK. In addition, although the study's cross-sectional design enabled a large sample to be recruited, this also prevented causal effects and mechanisms from being investigated. Finally, a pragmatic approach was adopted when choosing the questions included in the survey. Specifically, a small number of questions were selected that align with national surveys, thus enabling comparisons with the general population, while also minimising participant burden. Therefore, questionnaires that are more frequently used in this population (for example the Fagerström Test for Nicotine Dependence),^26^ which are based on theoretical frameworks regarding motivation (such as theory of planned behaviour),^27^ were not utilised. Consequently, although the pragmatic approach enabled national comparisons and extensive recruitment within a typically ‘hard-to-reach’ population, the adoption of such an approach limited the validity of the utilised questions.

### Future research

The sample has been embedded within recent studies examining the impact of pandemic restrictions on people with SMI.^24,25^ This demonstrates the potential of having a ‘research ready’ cohort of participants, who can be quickly recruited to future research projects. Consequently, a promising next step could be to recruit a sample of people with SMI to be part of an established longitudinal cohort, allowing causal processes and effects to be investigated and enabling more targeted interventions to be developed. Moreover, future research should prioritise prospectively investigating whether facilitating the perception of healthy living as being important is associated with people with SMI becoming more motivated to engage in healthier behaviours. Finally, although this study has identified factors that could be targeted to increase motivation to reduce health-risk behaviours, further research is needed to identify strategies to help people with SMI to act on this motivation and change health-risk behaviours. For example, research is required to examine how best to support people who are motivated to change to access existing interventions (for example smoking cessation apps, such as the NHS Stop Smoking App), along with investigations of the barriers that limit such access (for example lack of internet access or digital skills to be able to effectively use mobile apps^28^).

## Data Availability

The data that support the findings of this study are available from the corresponding author, E.P., upon reasonable request.

## Supplementary material

For supplementary material accompanying this paper visit https://doi.org/10.1192/bjp.2022.143. click here to view supplementary material
