# Supplementary material for: Health-risk behaviours among people with severe mental ill health: understanding modifiable risk in the Closing the Gap Health Study
Source: Br J Psychiatry. 2023 Apr;222(4):160–6. doi: 10.1192/bjp.2022.143 (PMC10895492; doi:10.1192/bjp.2022.143)
Supplement: Supplementary file 1 [file S000712502200143Xsup.zip › S000712502200143Xsup001.docx]

**Supplementary Appendix 1**

**Measures**

***Demographic Information***

Socio-demographic characteristics gathered from participants included age, gender, employment status, ethnicity, and neighbourhood deprivation. Gender was categorised as ‘male’, ‘female’, ‘transgender’, and ‘preferred not to say’. Employment status was dichotomised so that participants were distinguished between ‘being in paid employment’ and ‘not being in paid employment’. Ethnicity was categorised as ‘White’, ‘South Asian’, ‘Other Asian’, ‘Black’, ‘Mixed’, and ‘Other’; however, for the application of regression analyses ethnicity was dichotomised into either ‘White’ or ‘Non-White’. Each participant’s level of neighbourhood deprivation was determined by linking their home postcode to the English Index of Multiple Deprivation, which is a geographic measure of relative deprivation. The IMD ranks each area from the most to least deprived, based on seven domains (e.g., quality of local environment, crime, barriers to services, etc.). The indices are aggregated into decile groups (where 1 = most deprived, 10 = least deprived).

***Health Information***

Participants were asked to self-report their height and weight, and this information was used to calculate each participant’s Body Mass Index (BMI). This was then applied to categorise each participant as being either underweight (less than 18.5), healthy weight (18.5-24.5), overweight (24.5-30), or obese (over 30), (https://www.nhs.uk/common-health-questions/lifestyle/what-is-the-body-mass-index-bmi/). Participants were also asked to rate their general health in the last 12 months as being either ‘excellent’, ‘good’, ‘moderate’, ‘poor’, or ‘very poor’, and if any health problem limited their activity.

***Perceived Importance of Health***

Each participant’s self-perceived importance of maintaining a healthy lifestyle was ascertained through the following question: “how important is it that you maintain a healthy lifestyle?”. Response options that were available included ‘a top priority’, ‘moderately important’, and ‘I don’t worry about it’. Responses were then dichotomised into either ‘some importance’ (responses of ‘a top priority’ or ‘moderately important’) or ‘no importance (responses of ‘I don’t worry about it’).

***Health Risk Behaviours^[[1]](#footnote-1)^***

**Physical Activity.** Self-reported levels physical activity was ascertained with the question: “In general, how often do you take part in any sport or physical activity?”. Response options included ‘every day’, ‘every other day’, ‘at least once a week’, ‘more than once a month but less than once a week’, ‘less than once a month’, and ‘never’. Frequency of physical activity was then categorised as either: ‘daily or every day’ (responses of ‘every day’ or ‘every other day’); ‘weekly’ (responses of ‘at least once a week’); ‘less than weekly’ (responses of ‘more than once a month but less than once a week’ or ‘less than once a month’); or ‘never’ (responses of ‘never’).

**Diet.** Each participant’s consumption of fruit and vegetables was measured with the question: “in general, how many portions of fruit and vegetables do you eat per day?”. Response options included ‘I don’t eat fruit and vegetables’, ‘one’, ‘two’, ‘three’, ‘four’, and ‘five or more’. A dichotomous variable was derived for ‘meeting current guidelines for consumption of fruit and vegetables’, with those who reported eating five or more portions being classed as meeting guidelines, and all other responses being categorised as ‘not meeting current guidelines’ (<https://www.nhs.uk/live-well/eat-well/the-eatwell-guide/>).

**Smoking.** Each participant’s smoking status was assessed by the question ‘do you smoke?’. Participants could respond either ‘yes’, ‘no I have never smoked’, or ‘no but I used to smoke’. Participants were also asked how many cigarettes they smoked per day, with the following response options being available: ‘none’, ‘1-9’, ’10-19’, and 20 or more’. A variable for heaviness of smoking was derived from this information. Participants who reported that they currently smoke but then answered 0 for number of cigarettes they smoked per day were classed as ‘occasional smokers’, participants who reported smoking 1-9 or 10-19 cigarettes per day were classed as regular smokers, while participants who reported smoking 20 or more cigarettes per day were classed as heavy smokers.

***Motivations to Change Behaviours***

Participants’ levels of motivation to change specific health risk behaviours were measured using single items for the behaviours of physical activity, consumption of fruit and vegetables, and smoking. All participants were asked “would you like to take more exercise?” and “would you like to change your diet or lose some weight?”, while only those who reported smoking were asked ‘would you like to cut down or quit smoking?”. Participants could respond to each question with either ‘yes’, ‘no’, or ‘don’t know’. Responses were then dichotomised into either ‘wanting to change’ (responses of ‘yes’) or ‘not wanting to change or unsure (responses of ‘no’ or ‘don’t know’).

1. It was initially intended to also investigate alcohol consumption as an additional health risk behaviour, with participants being asked three questions related to their alcohol use. However, many participants did not answer these questions (~19%), and it was deemed that missing values were not missing at random. Therefore, alcohol consumption was not examined further, as it was deemed inappropriate with the available data. [↑](#footnote-ref-1)
