# Supplementary material for: Health-risk behaviours among people with severe mental ill health: understanding modifiable risk in the Closing the Gap Health Study
Source: Br J Psychiatry. 2023 Apr;222(4):160–6. doi: 10.1192/bjp.2022.143 (PMC10895492; doi:10.1192/bjp.2022.143)
Supplement: Supplementary file 1 [file S000712502200143Xsup.zip › S000712502200143Xsup002.docx]

| **Supplementary Table 1.**  ***Factors Associated with Importance of Maintaining a Healthy Lifestyle (n=6138) – Complete Case Analysis*** | | |
| --- | --- | --- |
| **Variable** | **Odds Ratio (95% CI)** | **p** |
| Gender |  |  |
| Male | 1 |  |
| Female | 1.46 (1.25-1.69) | <0.001* |
| Age | 1.00 (1.00-1.01) | 0.163 |
| Ethnicity |  |  |
| White | 1 |  |
| Non-White | 1.29 (1.02-1.66) | 0.035 |
| Employment |  |  |
| In Paid Employment | 1 |  |
| Not In Paid Employment | 0.69 (0.55-0.85) | <0.001* |
| Index of Multiple Deprivation | 1.07 (1.04-1.10) | <0.001* |
| Body Mass Index |  |  |
| Healthy Weight | 1 |  |
| Underweight | 0.68 (0.43-1.12) | 0.119 |
| Overweight | 1.03 (0.85-1.24) | 0.789 |
| Obese | 0.96 (0.80-1.15) | 0.649 |
| General Health Rating |  |  |
| Excellent/Good | 1 |  |
| Moderate | 0.68 (0.57-0.82) | <0.001* |
| Poor/Very Poor | 0.45 (0.37-0.55) | <0.001* |
| *Statistically significant when tested against a Bonferroni-adjusted alpha value of 0.0125. | | |
