# Supplementary material for: Health-risk behaviours among people with severe mental ill health: understanding modifiable risk in the Closing the Gap Health Study
Source: Br J Psychiatry. 2023 Apr;222(4):160–6. doi: 10.1192/bjp.2022.143 (PMC10895492; doi:10.1192/bjp.2022.143)
Supplement: Supplementary file 1 [file S000712502200143Xsup.zip › S000712502200143Xsup003.docx]

| **Supplementary Table S2**  ***Factors Associated with Motivation to Take More Exercise (n=3615), Change Diet or Lose Weight (n=5585), or Cut Down or Quit Smoking (n=2415) – Complete Case Analyses*** | | | | | | |
| --- | --- | --- | --- | --- | --- | --- |
|  | **Take More Exercise** | | **Change Diet/Lose Weight** | | **Cut Down/Quit Smoking** | |
| **Variable** | **Odds Ratio (95% CI)** | **p** | **Odds Ratio (95% CI)** | **p** | **Odds Ratio (95% CI)** | **p** |
| Gender |  |  |  |  |  |  |
| Male | 1 |  | 1 |  | 1 |  |
| Female | 1.38 (1.19-1.61) | <0.001* | 2.09 (1.86-2.36) | <0.001* | 1.22 (1.03-1.46) | 0.024 |
| Age | 0.98 (0.98-0.99) | <0.001* | 1.05 (1.03-1.08) | <0.001* | 1.00 (0.99-1.00) | 0.258 |
| Age^2^ | - | - | 1.00 (1.00-1.00) | <0.001* | - | - |
| Ethnicity |  |  |  |  |  |  |
| White | 1 |  | 1 |  | 1 |  |
| BME | 0.94 (0.75-1.18) | 0.582 | 0.97 (0.81-1.16) | 0.723 | 1.08 (0.84-1.39) | 0.550 |
| Employment |  |  |  |  |  |  |
| In Paid Employment | 1 |  | 1 |  | 1 |  |
| Not In Paid Employment | 0.50 (0.39-0.62) | <0.001* | 0.85 (0.72-0.99) | 0.042 | 0.86 (0.67-1.11) | 0.256 |
| Index of Multiple Deprivation | 1.03 (1.01-1.06) | 0.015 | 1.02 (1.00-1.04) | 0.094 | 1.01 (0.98-1.04) | 0.533 |
| General Health Rating |  |  |  |  |  |  |
| Excellent/Good | 1 |  | 1 |  | 1 |  |
| Moderate | 1.12 (0.93-1.34) | 0.237 | 1.28 (1.12-1.47) | <0.001* | 1.19 (0.98-1.46) | 0.083 |
| Poor/Very Poor | 1.33 (1.10-1.62) | 0.004* | 1.41 (1.21-1.64) | <0.001* | 1.34 (1.08-1.66) | 0.007* |
| Importance of Healthy Lifestyle |  |  |  |  |  |  |
| No Importance | 1 |  | 1 |  | 1 |  |
| Some Importance | 2.85 (2.37-3.42) | <0.001* | 1.80 (1.54-2.10) | <0.001* | 1.66 (1.35-2.04) | <0.001* |
| Body Mass Index |  |  |  |  |  |  |
| Healthy Weight | 1 |  | - | - | 1 |  |
| Underweight | 1.38 (0.81-2.40) | 0.247 | - | - | 0.70 (0.41-1.18) | 0.175 |
| Overweight | 1.35 (1.11-1.63) | 0.002* | - | - | 1.08 (0.88-1.34) | 0.448 |
| Obese | 1.72 (1.43-2.07) | <0.001* | - | - | 1.23 (1.00-1.50) | 0.045 |
| Frequency of Physical Activity |  |  |  |  |  |  |
| Weekly | 1 |  | - | - | - | - |
| Less Than Weekly | 1.06 (0.87-1.29) | 0.576 | - | - | - | - |
| Never | 0.59 (0.49-0.70) | <0.001* | - | - | - | - |
| Heaviness of Smoking |  |  |  |  |  |  |
| ≥20 Cigarettes Per Day | - | - | - | - | 1 |  |
| <20 Cigarettes Per Day | - | - | - | - | 1.06 (0.89-1.26) | 0.524 |
| *Statistically significant when tested against a Bonferroni-adjusted alpha value of 0.0125 (0.05/4). | | | | | | |
